# Leucine-rich repeat kinase 2 limits dopamine D1 receptor signaling in striatum and biases against heavy persistent alcohol drinking

**DOI:** 10.1038/s41386-023-01731-z

**Published:** 2023-09-08

**Authors:** Daniel da Silva, Aya Matsui, Erin M. Murray, Adamantios Mamais, Michael E. Authement, Jung Hoon Shin, Marlisa Shaw, Dorit Ron, Mark R. Cookson, Veronica A. Alvarez

**Affiliations:** 1 Laboratory on Neurobiology of Compulsive Behaviors, National Institute on Alcohol Abuse and Alcoholism, NIH, Bethesda, MD 20892 USA; 2 Cell Biology and Gene Expression Section, Laboratory of Neurogenetics, National Institute on Aging, NIH, Bethesda, MD 20892 USA; 3 Department of Neurology, University of California San Francisco, San Francisco, CA USA; 4 Center on Compulsive Behaviors, Intramural Research Program, NIH, Bethesda, MD 20892 USA; 5 Present Address: NIMH, National Institutes of Health, Bethesda, USA

**Keywords:** Addiction, Neuroscience

## Abstract

The transition from hedonic alcohol drinking to problematic drinking is a hallmark of alcohol use disorder that occurs only in a subset of drinkers. This transition requires long-lasting changes in the synaptic drive and the activity of striatal neurons expressing dopamine D1 receptor (D1R). The molecular mechanisms that generate vulnerability in some individuals to undergo the transition are less understood. Here, we report that the Parkinson’s-related protein leucine-rich repeat kinase 2 (LRRK2) modulates striatal D1R function to affect the behavioral response to alcohol and the likelihood that mice transition to heavy, persistent alcohol drinking. Constitutive deletion of the *Lrrk2* gene specifically from D1R-expressing neurons potentiated D1R signaling at the cellular and synaptic level and enhanced alcohol-related behaviors and drinking. Mice with cell-specific deletion of *Lrrk2* were more prone to heavy alcohol drinking, and consumption was insensitive to punishment. These findings identify a potential novel role for LRRK2 function in the striatum in promoting resilience against heavy and persistent alcohol drinking.

## Introduction

Alcohol use disorder (AUD) is a chronic relapsing disorder characterized by an inability to stop alcohol use despite adverse consequences [1]. Loss of control over alcohol drinking leads to abuse and hinders long-term abstinence, driving relapse. Only a fraction (~8%) of those who consume alcohol are diagnosed with AUD each year [2], pointing to the existence of risk and resilience factors for developing the disorder [3, 4]. The striatum plays a central role in learning and execution of reward-motivated behaviors, making this brain region very relevant for substance use disorders. In fact, functional and morphological alterations in the striatal circuitry have been linked to AUD [5] and we hypothesize that difference in the striatal circuitry can drive vulnerability for developing AUD.

The leucine-rich repeat kinase 2 (*Lrrk2)* gene is highly expressed in the striatum and plays an important role in regulating synapse formation and synaptic transmission [6, 7]. Mutations in the human *LRRK2* gene are associated with Parkinson’s disease [8, 9], which is characterized by prominent impairment in dopamine signaling and basal ganglia function. Recent work has linked changes in striatal expression of the *Lrrk2* gene with alcohol drinking in humans and rodents [10, 11]. A positive correlation between striatal levels of *Lrrk2* mRNA and alcohol drinking was reported in mice, especially an association with inflexible alcohol drinking that persists despite adverse outcomes or punishment [10]. However, it remains unclear whether this association is causal, and which are possible mechanisms underlying the impact of LRRK2 activity on alcohol reinforcement.

LRRK2 was shown to act as a negative modulator of dopamine D1 receptors (D1R) within the striatum. Mutations in *Lrrk2* alter membrane trafficking and surface expression of D1R [12, 13]. Global deletion of *Lrrk2* enhanced protein kinase A signaling downstream of D1R and altered dendritic spine morphology and synaptic strength in striatal medium spiny neurons of developing mice [14, 15]. Gain-of-function mutations of *Lrrk2* were shown to reduce PKA activity [15]. Thus, LRRK2 is proposed to limit D1R signaling via PKA pathway in the direct-pathway medium spiny neurons of the striatum.

In the striatum, D1R are expressed in the direct pathway medium spiny neurons (D1- MSNs) and regulate the reinforcing properties of alcohol. Deletion of the gene encoding for D1R (*Drd1a*) impairs alcohol drinking and preference [16]. Pharmacological blockade of D1-like receptors, but not D2-like receptors, attenuates alcohol consumption when delivered in the dorsomedial striatum [17–19]. Further, D1-like antagonists impair alcohol-seeking in an operant task [20]. Other evidence shows that alcohol exposure elicits functional and structural plasticity selectively in D1-MSNs. For example, a single alcohol drinking session potentiates synaptic drive onto D1- MSNs in the nucleus accumbens [18] and repeated cycles of alcohol exposure induce a long-lasting potentiation in excitatory synaptic transmission in D1-MSNs in the dorsomedial striatum, but not in D2-MSNs [17]. We hypothesize that AUD vulnerability could arise in part from enhanced D1R function.

More specifically, this study tests the hypothesis that loss of LRRK2 function in D1R expressing neurons promotes PKA signaling and D1R activation, impacting on the excitability of striatal direct-pathway D1-MSNs and the response to alcohol. We used mice carrying conditional *Lrrk2* alleles to generate cell-specific deletion of the *Lrrk2* gene in D1R expressing cells and evaluated the receptor function and alcohol-related behaviors. The results showed that selective deletion of *Lrrk2* in D1R expressing cells, but not other cells such as D2-expressing cells, potentiates the cellular and behavioral response to D1-like receptor agonist and alcohol. Loss of *Lrrk2* in D1R expressing neurons promoted heavy and punishment-resistant alcohol drinking. The results indicate that low activity of LRRK2 in direct-pathway neurons could become a vulnerability factor for out-of-control alcohol drinking. We propose that other factors that enhance LRRK2 activity in D1-MSNs will limit dopamine signaling via D1R and confer resilience against heavy alcohol drinking and drinking despite adverse consequences.

## Materials and methods

### Animals

All experiments were approved and performed in accordance with guidelines from the Animal Care and Use Committee from National Institute on Alcohol Abuse and Alcoholism, NIH. Male and female mice (8–18-week-old) were used in all experiments. Data from both male and female subjects were combined for analysis in most experiments. In those experiments specifically powered to detect potential sex differences, sex was treated as a biological variable. In such cases, the data was analyzed separately for males and females, and individual plots were generated to visualize any potential divergences between the sexes. Lrrk2^loxP/loxP^ mice were generated [21] and generously provided by Dr. Huaibin Cai (NIA, NIH). Drd1a-cre (B6.FVB(Cg)- Tg(Drd1-cre)EY262Gsat/Mmucd) and Adora2a-cre (B6.FVB(CG)-TG(ADORA2A- CRE)KG139GSAT/MMUCD) mouse lines were used for cell-specific deletion. A Global-Lrrk2- KO mouse (B6.129×1(FVB)-LRRK2TM1.1CAI/J) and a D1Td tomato reporter line (B6.Cg-

Tg(Drd1a-tdTomato)6Calak/J) were used for specific experiments as detailed below. For all experiments using transgenic mice, Cre-negative Lrrk2^loxP/loxP^ littermates were used as controls. Mice were genotyped by Transnetyx (Cordova, TN). Mice were grouped-housed under normal light cycle (12 h dark/light, lights on 6:30 am). For alcohol drinking experiments, mice were singled-housed and transferred to reverse light cycle (12 h dark/light, lights off 6:30 am) at least 10 days before the start of the experiments. Standard rodent chow and water were always available *ad libitum* in home cage, except during DID sessions when water was removed for 4 h/session. No food deprivation was used in any experiment.

### Tissue and cell-specific quantification of Lrrk2 mRNA

RNA was extracted from dorsal striatum and lung samples using RNAeasy mini kit (Qiagen) and cDNA was synthetized using iScript (BioRad) according to manufacturer instructions. qPCR was performed using TaqMan Fast Advanced Master Mix (Thermo Fisher Scientific) with probes Mm01304130_m1 and Mm99999915_g1. Relative quantification was calculated according to ΔΔCt method and normalized by Gapdh expression and by control levels. For RNAscope, reagents, probes, and equipment from Advanced Cell Diagnostics were used. Coronal sections (16 µm) were sliced on cryostat at −20 °C. Brain slices containing striatum (~AP: +1.1 mm from Bregma) were processed according to manufacture protocol and mounted on Superfrost slides using ProLong™ Gold Antifade (Thermo Fisher Scientific).

### LRRK2 immunostaining

Immunostaining was performed using primary antibody [MJFF2 (c41-2)] (1:50, Abcam, #ab133474, produced recombinantly for high batch-to-batch consistency) and secondary antibody (Alexa Fluor® 488, Abcam, #ab150077, 1:1000) according to previously published protocol [22].

### c-Fos immunostaining

Mice expressing tdTomato under the Drd1a promoter were injected with either SKF81297 (2 mg/kg) or saline (10 ml/kg) 90 min prior to transcardial perfusion with 4% PFA. The detailed method for immunostaining and cell quantification is presented in the supplementary files.

### Electrophysiology

Mice (8–12 weeks-old) were anesthetized with isoflurane and perfused transcardially with warm artificial cerebrospinal fluid (aCSF) containing (in mM): 124 NaCl, 2.5 KCl, 1.3 MgCl_2_, 2.5 CaCl_2_, 1.0 NaH_2_PO_4_, 26.2 NaHCO_3_, 20 D-glucose, 0.4 ascorbate and 3 kynurenic acid. Sagittal brain slices (230 µm) were incubated in warm oxygenated aCSF for at least 30 min and moved to room temperature until used. Details about whole-cell patch clamp recordings and data analysis are presented in the supplementary files.

### Alcohol- and SKF81297 inducing locomotion

Locomotor activity was measured during the animal’s light cycle in a clear polycarbonate chamber (20 × 17 × 28) equipped with infrared photobeam detectors (Columbus Instruments). Baseline locomotor was recorded for one-hour followed by an extra hour of recordings following intraperitoneal injections of saline, alcohol or SKF81297 at the appropriate dose. Data was analyzed as beam breaks per 5 min and normalized by the third day of saline injection. For SKF81297 tests, locomotor activity was assessed using the IR actimeter system (Panlab).

### Open field and novel object exploration

Exploratory behavior was measured in an open-field acrylic/PVC chamber (40 ×40 × 40 cm). Mice were allowed to explore for 30 min and then returned to homecage for 5 min, during which time a novel object was placed in the center arena. Animals were placed back into the chamber and allowed to explore for an additional 15 min. The mouse position was tracked over time to estimate distance traveled using EthoVision XT (Noldus). Exploration was quantified based on active interactions with the object, specifically defined as instances when the mouse’s nose was directed towards the object and positioned within a proximity of 3 cm from the object.

### Loss of righting reflex

Mice were injected with alcohol (3 g/kg, 20 mL/kg, i.p.) and placed in the supine position in a v-shaped trough. Loss of righting reflex (LORR) was defined as the mouse’s inability to right itself three times within a period of 30 s. If mice failed to respond within 60 min, the session was terminated and the regain time was defined to 60 min. Blood samples were collected at the time of regaining righting reflex and blood alcohol concentration (BAC) was measured using AM1 Alcohol Analyser (Analox).

### Alcohol sensitization

The sensitization protocol was adapted from a previously published procedure [23]. Mice were first habituated to saline injections for two consecutive days then injected with alcohol (2 g/kg, 12.5 ml/kg) for 8 consecutive days. Locomotor activity was recorded for 15 min. Following the last day of alcohol injections, mice were left undisturbed in their home cage for 8 days followed by a single alcohol challenge session. Data was acquired using IR actimeter system (Panlab).

### Alcohol drinking behavior

#### Intermittent access two-bottle choice

Mice had intermittent access (Mon-Wed-Fri) to one bottle with unsweet 20% alcohol solution in tap water and continuous access to tap water in the home- cage for 4 weeks as previously described [24].

#### Alcohol dose-response and quinine adulteration

Mice had continuous access to alcohol and water 4 days/week for 7 weeks. Alcohol concentration was 20% during weeks 1–2 decreased to 10% and 5% during weeks 3–4. On week 5 and 6, 20% alcohol was adulterated with 0.25 or 0.5 mM quinine, respectively, on days 3–4. On week 7, water was adulterated with 0.25 mM or 0.5 mM quinine.

#### Drinking in the dark (DID)

a modified drinking in the dark procedure was used according to previously published [25]. Following DID procedure, mice were trained to self-administer alcohol in operant chambers.

#### Operant alcohol self-administration

the SIPPER training procedure was adapted from previously published study [25]. Training boxes were equipped with two levers (active and inactive), cue light (active ON unless during reward delivery) and a hole covered by a guillotine-style door that would open during sipper tube extension. Training sessions lasting 6 h, began about 3 h into the dark cycle, occurring every other day. Initially, mice were trained at FR1 for 9 sessions and then at FR3 for 4 additional sessions. Completion of the fixed ratio triggered the opening of the door and the extension of the sipper tube allowing access to 20% alcohol solution for 60 s. Contacts to the sipper tube were recorded. Presses on the inactive lever were recorded but had no consequence. A food pellet was available during all sessions. Mice not meeting the minimum criteria (>0.1 g/kg alcohol/day) were excluded from further analysis. PR session was modified from previously published study [26]. In addition to successfully completing the appropriated response ratio, the progress to the next ratio was also contingent to a minimum of 10 lick contacts to the sipper.

Session ended after 1 h of unsuccessful ratio progression or after a total of 5 h. Two quinine sessions were carried out as training sessions, except that the alcohol solution was adulterated with 0.25 mM quinine. Three foot-shock sessions were carried out, where alternate alcohol access was paired with a 0.5-s foot shock, with increasing intensities from 0.2 to 0.6 mA in separate sessions. Shock delivery was paired with the extension of the sipper tube. Resistance to punishment (quinine and shock session) was measured as the percentage change in alcohol consumption during the punished sessions compared to baseline levels. Licks were not recorded during foot shock sessions due to shared ground between the lickometer and shock scramble apparatus.

### Sucrose and sucralose preference test

Mice had continuous ad libitum access to water and sucrose for 6 h/day. Cohort 1 consisted of access to 1% sucrose solution on days 1 and 2, and 2% on days 3 and 4. Cohort 2 had access to 0.5% sucrose on days 5 and 6. Sucralose preference was assessed in a similar fashion as the sucrose test. Sucralose concentrations were 1%, 0.5% and 0.05%, respectively.

### Food consumption

Food consumption was recorded using regular chow (NIH 31, 4.7 kcal% fat; 3.0 Kcal/g) and high fat diet (D12492, 60 kcal% fat; 5.2 kcal/g; Research Diets, Inc). Mice had 24 h/day ad libitum access to regular chow for one week followed by HFD access for 2 weeks. Food was weighed every day at 10:00 am and mice were weighed on the seventh day of each week. Caloric intake was calculated by multiplying the amount of food consumed by the caloric content of each diet.

### Hot plate test

Mice were habituated for 10 min to the apparatus then placed on the metal surface maintained at a constant temperature of 52.2 °C. The time taken to elicit licking of the forepaws was recorded as the latency and used to score pain sensitivity. Each mouse was tested three times with a minimum intertrial interval of 10 min. Latency in each trial was averaged to calculate individual scores.

### Shock threshold sensitivity

Mice were habituated for 10 min to the operant chamber. The test consisted of delivery of multiple shocks of increased intensity, ranging from 0.2 to 0.8 mA in 0.1 mA increments every 30 s. Threshold was determined as the lowest shock intensity that elicited a startle response, defined as instances when the mouse jumped with all four paws out of the grid floor.

### Drugs and chemicals

the complete list of drugs is presented in the supplementary files.

### *Lrrk2* mRNA expression and Stereotaxic virus injections

*see* supplementary files.

### Statistical Analysis and Quantification

Graphs and analyses were performed in Prism 7 (GraphPad), Igor Pro 9 (Wavemetrics) and R software (version 4.0.3). Detailed statistical results can be found in the relevant figure legends and in supplementary Table S1. Results were considered significant at an alpha of 0.05. Data are presented as mean ± SEM.

## Results

### *Lrrk2* expression is enriched in MSNs in the striatum

**Fig. 1:**
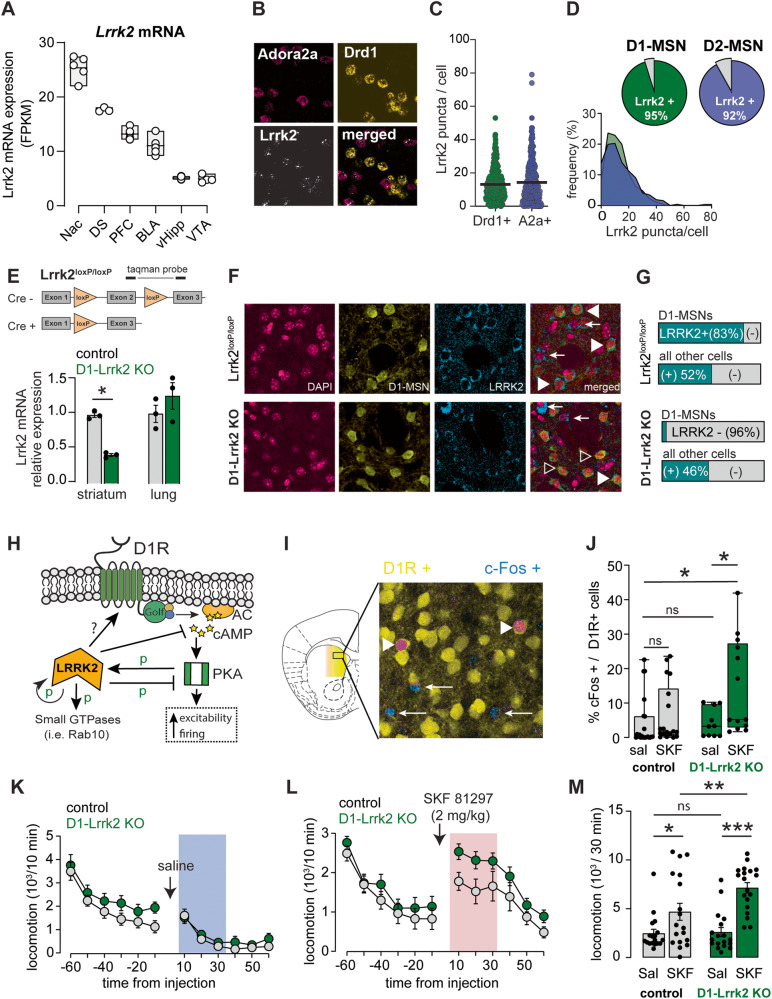
Targeted deletion of *Lrrk2* to D1R-expressing neurons potentiates the behavioral and cellular response to D1R activation. **A** *Lrrk2* mRNA expression across brain nuclei of the mesolimbic cortical circuit. NAc, nucleus accumbens; DS, dorsal striatum; PFC, prefrontal cortex; BLA, basolateral amygdala; vHipp, ventral hippocampus; VTA, ventral tegmental area. **B** Confocal images from DMS showing fluorescent RNA in-situ hybridization for *Lrrk2* (white), Adora2a (pink), and Drd1a mRNA (yellow). **C** Quantification of *Lrrk2* mRNA puncta per cell (Mann–Whitney *U* = 42802, *P* = 0.74). **D** Top, Percentage of D1R-positive and A2a- positive cells expressing *Lrrk2* mRNA. Bottom, frequency distribution of *Lrrk2* mRNA puncta in D1R- positive and A2a-positive cells. **E** *Top*, Conditional *Lrrk2* allele showing the LoxP insertion site. Bottom, Quantification of Lrrk2 mRNA in striatum (*t*_(4)_ = 14.5  *P* < 0.001) and lung (*t*_(4)_  = 1.13, *P* = 0.32). **F** Immunostaining for endogenous LRRK2 protein (blue), tdTomato in D1-MSNs (yellow), and DAPI (pink) in DMS sections. Filled arrowheads point to cells co-labeled with LRRK2 and tdTomato; open arrowheads point to cells labeled with tdTomato and negative for LRRK2; arrows point to cells labeled with LRRK2 and negative for tdTomato. **G** Degree of LRRK2 co-labeled with D1-MSNs or D1-negative cells (X^2^_(1398)_ = 249, *P* < 0.0001). **H** Modulation of D1R function by LRRK2. Golf, guanine nucleotide-binding protein G(olf) subunit alpha; cAMP, cyclic adenosine monophosphate; AC, adenylate cyclase; PKA, protein kinase A. **I** Confocal image of DMS from D1-Lrrk2-KO mouse showing D1-MSN in yellow and c-Fos in blue following injection of SKF81297. Arrows point to c-Fos positive cells and arrowheads to double positive cells. **J** Percentage of double positive cells over total D1-MSN after systemic administration of saline or SKF81297 (Aligned Rank Transformed ANOVA [ART]: no genotype effect: *F*_(1,59)_ = 0.9, *P* = 0.34; interaction: *F*_(1,59)_ = 4.0, *P* < 0.05; ART contrasts test *P* < 0.05 for D1-Lrrk2-KO saline vs SKF and control saline vs D1-Lrrk2-KO SKF). **K** Basal locomotion measured before and after saline injection (pre- injection: *F*_(1,35_) = 2.3, *P* = 0.13; post-injection: *F*_(1,35)_ = 0.79, *P* = 0.38). **L** Locomotion measured before and after SKF81297. **M** Average locomotion during the initial 30 min post injection (REML, SKF: *F*_(1,34)_ = 34.8; genotype: *F*_(1,3)_ = 4.3; interaction: *F*_(1,34)_ = 4.2, *P* < 0.05; Sidak’s test *P* < 0.01 for SKF control vs SKF D1-Lrrk2-KO). For all panels, data from Lrrk2^loxP/loxP^ is shown in gray and from D1-Lrrk2-KO in green; bars represent mean ± S.E.M and symbols represent values from individual mice. (*) denotes *P* < 0.05, **P < 0.01, ***P < 0.001.

Using publicly available RNA-seq dataset [27–29], we found that *Lrrk2* mRNA is remarkably enriched in the striatum compared to other limbic regions, such as the hippocampus, prefrontal cortex, basolateral amygdala, and ventral tegmental area (Fig. 1A). Our own RNA in situ hybridization experiments revealed that *Lrrk2* mRNA is expressed at similar levels in D1-MSNs (Drd1a+) and D2-MSNs (Adora2a+), and that the majority of these neurons express *Lrrk2* mRNA in the DMS (Fig. 1B–D).

### Cell-specific constitutive deletion of *Lrrk2* potentiates D1R-like function in D1-MSNs

We probed how LRRK2 activity regulates D1R function and alcohol-related behaviors by generating a mouse line with constitutive deletion of the *Lrrk2* gene from D1R-expressing cells. Mice bearing conditional alleles for the *Lrrk2* gene (Lrrk2^loxP/loxP^) were crossed with mice expressing Cre recombinase under the *Drd1a* promoter (D1-Lrrk2-KO, Fig. 1E). Regional and cellular specificity of *Lrrk2* deletion was assessed at the mRNA and protein level. D1-Lrrk2-KO showed reduced *Lrrk2* mRNA levels in the striatum but not in the lungs (Fig. 1E). LRRK2 protein level was reduced in D1+ neurons and not affected in D1- neurons in the DMS (Fig. 1F, G).

We studied the intracellular signaling downstream of D1R activation (Fig. 1H) and observed increased c-Fos expression in D1-MSNs of D1-Lrrk2-KO mice following low-dose D1- like agonist administration (Fig. 1I). After control saline injection, there was no difference between the genotypes in the overall percentage of D1-MSNs labeled with c-Fos in the DMS (Fig. 1J). However, a low dose of the D1-like receptor agonist SKF81297 (2 mg/kg, i.p.) was sufficient to increase the percent of c-Fos expressing D1-MSNs in D1-Lrrk2-KO mice but not littermate controls (Fig. 1J). SKF81297 injection did not increase c-Fos expression in mice with postnatal deletion of the *Lrrk2* gene in the DMS (Fig S1A).

The behavioral impact of *Lrrk2* deletion was assessed by recording locomotor activity after injection of saline or SKF81297 (2 mg/kg, i.p.*)* in naïve mice of both genotypes. Baseline locomotion was similar between genotypes (Fig. 1K). SFK81297 administration increased locomotion in both genotypes (Fig. 1L, M), but D1-Lrrk2-KO showed larger locomotion during the first 30 min after injection (Fig. 1M).

### D1-like agonist increases excitability and synaptic transmission of D1-MSNs lacking *Lrrk2*

**Fig. 2:**
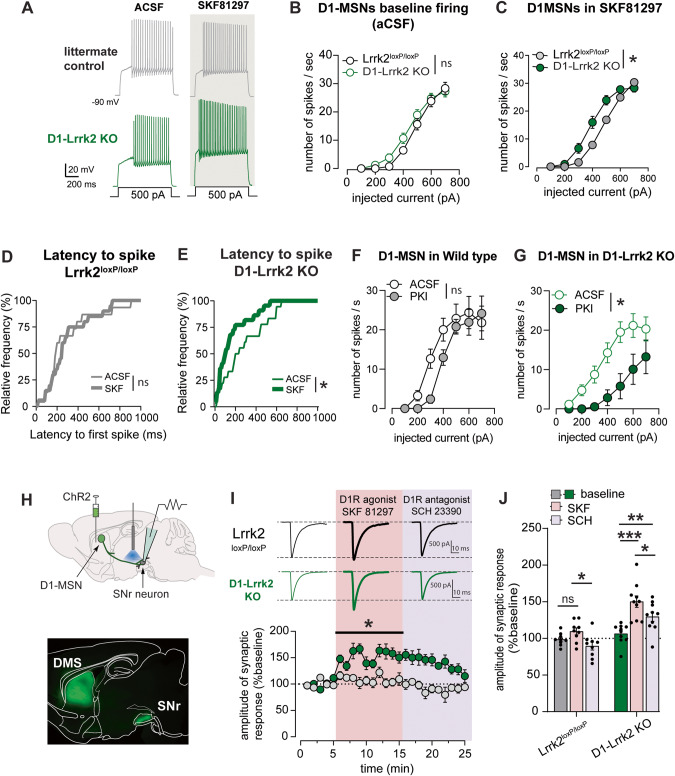
Deletion of *Lrrk2* in D1R-expressing neurons potentiates the electrophysiological response to D1-like agonist. **A** Representative traces of action potentials recorded from D1-MSNs in response to current step injection during whole-cell current-clamp recordings in control aCSF conditions and after incubation with SKF81297 (1 µM) in the DMS of D1-Lrrk2-KO (green) mice and littermate controls (gray). **B**, **C** Input-output curve of firing rate from D1-MSNs in response to current steps of increasing amplitude after incubation of slices with aCSF (**B**; no genotype effect: *F*_(1,55)_ = 1.5, *P* = 0.23; no interaction: *F*_(6,330)_ = 1.3, *P* = 0.26; *n* = 28–30 cells, 11/10 mice) or SKF81297 (**C**; genotype effect: *F*_(1,41)_ = 4.1; *P* < 0.05; interaction: *F*_(6,246)_ = 4.7, *P* < 0.0001; *n* = 21–22 cells, 7/7 mice). **D**, **E** Cumulative histogram of the latency to the first action potential at 500 pA current step for littermate controls (E; KS = 0.19, *P* = 0.16) and D1-Lrrk2-KO mice (F; KS = 0.3, *P* < 0.005). **F**, **G** Input-output curve of firing rate from D1-MSNs in response to current steps of increasing amplitude during baseline and after incubation of slices with PKA inhibitor PKI (1 µM) (F, no PKI effect: *F*_(1,20)_ = 1.4, *P* = 0.25; no interaction: *F*_(6,120)_ = 1.6, *P* = 0.15; *n* = 9 cells, 3 mice; G, PKI effect: *F*_(1,26)_ = 8.7, *P* < 0.01; interaction: *F*_(6,156)_ = 2.2, *P* = 0.04; *n* = 9 cells, 3). **H** Top, schematic diagram showing injection site of ChR2 viral vector in the DMS, the site of the recordings in the midbrain substantia nigra reticulata (SNr), and the local fiber optic used to stimulate direct-pathway axons in the SNr; Bottom, fluorescent image shows expression of ChR2 (green) in the DMS and labeled axons in the SNr. **I** Top, representative traces of voltage-clamp recordings from neurons in the SNr showing optogenetic-evoked synaptic response during baseline and after bath application of SKF81297 and SCH23390 in brain slices from D1-Lrrk2-KO (green) and littermate controls (gray); Bottom, time course of recorded inhibitory synaptic responses in putative GABAergic neurons of SNr in response to optogenetic stimulation of direct-pathway MSNs in the presence of SKF81297 (pink shaded area) or SCH23390 (purple shaded area). **J** Average normalized oIPSC amplitude (first 5 min) in response to SKF81297 and SCH23390 application (genotype: *F*_(1,17)_ = 34; treatment: *F*_(2,34)_ = 34; interaction: *F*_(2,34)_ = 6.4, *P* < 0.005; Sidak’s test: baseline vs SKF: *P* < 0.0001 for D1-Lrrk2-KO). For all panels, bars represent mean ± S.E.M and symbols represent values from individual slices. (*) denotes *P* < 0.05, (***) denotes *P* < 0.001.

Physiological readouts of D1R activation were recorded in genetically identified D1-MSNs, achieved by crossing with Drd1-tdTomato mice or by cre-dependent expression of fluorescent protein in mice expressing cre-recombinase under the Drd1a promoter (D1-Lrrk2-KO or Drd1a-Cre control). Recordings were made from labeled neurons in brain slices containing the DMS. In current-clamp mode, we measured action potentials and the excitability of the D1-MSNs under baseline conditions and after D1-like agonist SKF81297. The input-output curves at baseline were overlapping for the genotypes (Fig. 2A, B). However, after incubation with SKF81297 (1 µM), there was a leftward shift in the input-output curve from D1- Lrrk2-KO (Fig. 2A, C), indicating a lower threshold to fire spikes and higher excitability. This effect seems caused by a shortening of the latency to the first spike and increase in the input resistance in D1-Lrrk2-KO (Fig. 2E; S1B). SKF81297 had no significant effect on latency or input resistance in control mice (Fig. 2D; S1B). SKF81297 also had no effect on the afterhyperpolarization current in either genotype (Fig S1C). Preincubating brain slices with the PKA inhibitor PKI blocked the excitability increase caused by the D1-like agonist SKF81297 in D1-Lrrk2 KO mice (Fig. S1D). Interestingly, we observed a potent suppression of D1-MSN excitability by PKI in slices from D1-Lrrk2-KO, while control mice showed no effect (Fig. 2F, G). These findings indicate heightened regulation of neuronal excitability by PKA activity in mice with Lrrk2 deletion, supporting *Lrrk2*’s role as a negative regulator of PKA.

In voltage-clamp recordings, we also assessed the frequency and amplitude of spontaneous excitatory postsynaptic currents and AMPAR/NMDAR ratio of electrically evoked glutamatergic synaptic responses. All these parameters were similar between genotypes (Fig. S1E–G), suggesting no major changes in the density and strength of excitatory transmission onto D1- MSNs in adult D1-Lrrk2 KO mice, consistent to previous observations from adult global Lrrk2 KO mice [7].

To assess synaptic transmission from D1-MSNs to midbrain target neurons, we expressed Channelrhodopsin-2 in the DMS and recorded from neurons in substantia nigra pars reticulata (SNr). Optogenetic stimulation evoked synaptic responses in SNr neurons (Fig. 2H). Bath application of SKF81297 (1 µM) increased the amplitude of the synaptic responses (oIPSC) in D1- Lrrk2-KO mice (Fig. 2I, J). The increase was reversed by the D1-like antagonist SCH23390 (Fig. 2I, J). Furthermore, the agonist reduced the paired-pulse ratio of the synaptic responses in D1- Lrrk2-KO (Fig. S1H), suggesting D1R activation increases the probability of vesicle release.

### High concentration of alcohol exerts larger effect on D1-MSNs excitability in D1-Lrrk2-KO mice

**Fig. 3:**
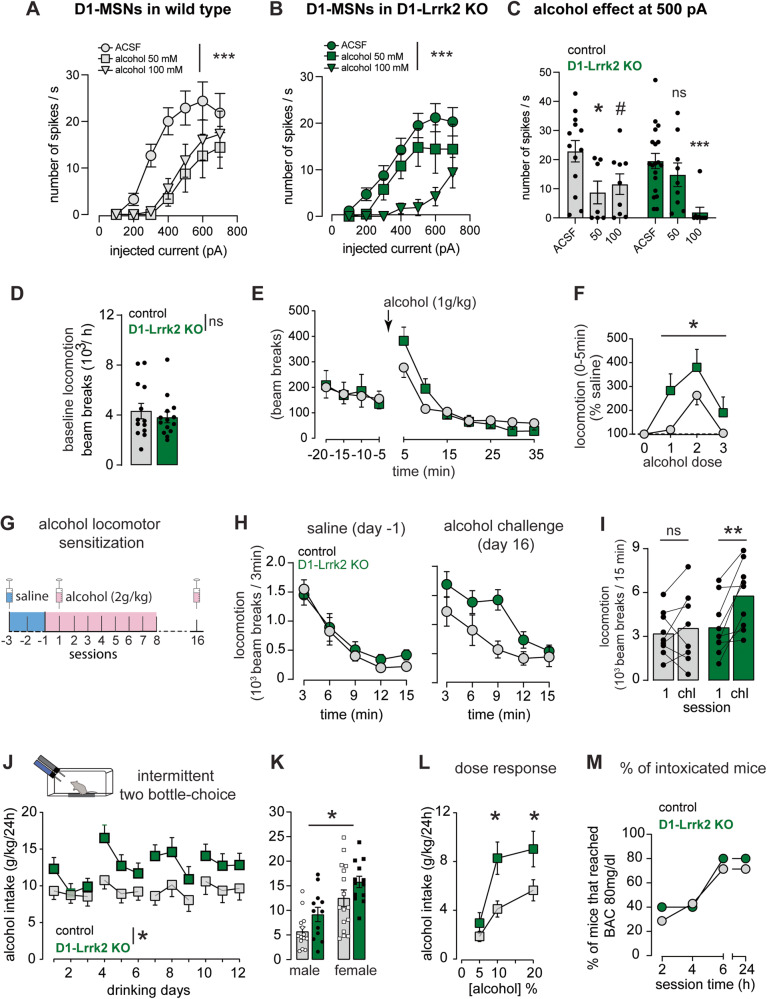
*Lrrk2* expression in D1-MSNs regulates alcohol stimulation, locomotor sensitization, and drinking. Input-output curve of firing rate from D1-MSNs in response to current steps of increasing amplitude after incubation of slices with alcohol (**A**, alcohol effect: *F*(2,26_)_ = 5.7, *P* < 0.0001; interaction: *F*_(12,156)_ = 2.1, *P* < 0.05; *n* = 8/9 cells, 5/3 ACSF/alcohol; **B** alcohol effect: *F*_(2,34)_ = 7.9, *P* = *P* < 0.0001; interaction: *F*_(12,204) _= 2.3, *P* < 0.01; *n* = 5/9 cells, 3/3 ACSF/alcohol). **C** Inhibition of D1-MSNs firing by alcohol at 500 pA current step (*F*_(2,60)_ = 10.4, *P* < 0.0001). **D** Basal locomotion during the habituation session (*t*_(25_) = 0.67, *P* = 0.51). **E** Locomotor activity before and after systemic administration of alcohol (1 g/kg; *F*_(1.79,51.9)_ = 15.3; *P* < 0.0001). **F** Dose-dependence of the locomotor response induced by systemic administration of alcohol during the first 5 min after alcohol injection (REML, genotype effect: *F*_(1,35)_ = 4.2, *P* < 0.05). **G** Graphical representation of the alcohol sensitization protocol. **H** Basal locomotion during habituation day (saline injection) in alcohol-naive mice *(left)* and during alcohol challenge day (*right*). **I** Mean locomotor activity during the first day of alcohol injection and the challenge day (REML: session effect: *F*_(1,15)_ = 9.8, *P* <  0.05 and interaction: *F*_(1,15) _= 4.9, *P* < 0.05; Sidak’s test, control: *P* = 0.78, D1-Lrrk2-KO: *P* < 0.005). **J** Mean alcohol intake (g/kg/24 h) during intermittent two-bottle-choice sessions (genotype effect: *F*_(1,53) _= 4.0, *P* < 0.05). **K** Sex differences in the overall average of alcohol intake during the 12 sessions of intermittent two-bottle- choice (genotype effect: *F*_(1,51)_ = 6.0; *P* < 0.05). **L** Alcohol intake as a function of the alcohol concentration offered (genotype effect: *F*_(1,21)_ = 5.5, *P* <  0.05; Tukey’s test, *P* < 0.05). **M** Percentage of mice that reached blood alcohol intoxication (>80 mg/dl) at 2, 4, 6, and 24 h after the beginning of the alcohol drinking session. For all panels, data from Lrrk2^loxP/loxP^ is shown in gray and from D1-Lrrk2-KO in green; bars represent mean ± S.E.M and symbols represent values from individual mice. (*) denotes *P* < 0.05; (**) denotes *P* < 0.005; (***) denotes *P* < 0.0001; (#) denotes *P* = 0.06.

The acute effect of alcohol on the D1-MSN excitability was assessed by incubating brain slices containing the DMS in 50 or 100 mM alcohol. Genetically identified D1-MSNs were recorded in current clamp mode, as described in Fig. 2. In slices from control mice, the application of alcohol 50 mM caused a downward shift in the excitability curve, leading to a reduction of firing by ~60% in D1-MSNs at 500 pA current injection (Fig. 3A, C). A higher alcohol concentration did not produce further inhibition, indicating a saturation in the alcohol effect on D1-MSN excitability. In contrast, alcohol 50 mM had no effect on firing in D1-Lrrk2-KO mice. However, higher alcohol concentration promoted a profound inhibitory effect, suppressing firing by ~90% (Fig. 3B, C).

### Deletion of *Lrrk2* promotes alcohol-induced stimulation but not ataxia

Mice with enhanced D1R function display higher locomotor stimulation and drinking preference for alcohol [30]. Here, we assessed the effect of three doses of alcohol (1, 2, and 3 g/kg i.p.) on locomotor activity. Basal locomotion after saline injections was similar between genotypes (Fig. 3D). Alcohol injection induced a dose-dependent increase in locomotion in both genotypes specifically within the first 5 min post-injection (Fig. 3E, F; S2D, E). Alcohol-induced ataxia was assessed using the LORR test. Alcohol naïve mice received 3 g/kg (i.p.) alcohol and the latency to lose and regain the righting reflex was recorded. Mice from both genotypes showed similar mean latency to LORR (Fig. S2A), and similar mean time to regain the righting reflex (Fig. S2B). The BAC was measured at the time of regaining the righting reflex and was similar in both genotypes (Fig. S2C).

### Loss of LRRK2 function enhances alcohol sensitization and drinking

Locomotor sensitization to alcohol is mediated by D1R activation in the striatum [23, 31]. We measured the development and expression of alcohol locomotor sensitization following 8 daily injections of alcohol (2 g/kg, i.p.; Fig. 3G). Basal locomotor activity was similar between genotypes (Fig. 3H-left) and daily alcohol injections promoted an increased locomotor response (Fig S2L). During the challenge test, D1-Lrrk2-KO mice showed a larger locomotor response to alcohol than controls (Fig. 3H-right, 3I). Additionally, locomotor response during the challenge test was larger than on day 1 specifically in D1-Lrrk2-KO mice (Fig. 3I).

Voluntary drinking and preference for alcohol were assessed using an intermittent two- bottle-choice paradigm. Mice from both groups showed a mild pattern of escalation on alcohol consumption as previously shown in the literature [32–34]. D1-Lrrk2-KO mice showed increased alcohol drinking (Fig. 3J, K) and preference (Fig S2F) compared to controls. Dose dependency on alcohol drinking was measured in an independent cohort of mice. Compared to controls, D1-Lrrk2- KO mice consumed more alcohol when given access to 10% and 20% alcohol solutions (Fig. 3L). Both genotypes reached 80 mg/dl during the 24 h drinking period (Fig. 3M). Alcohol consumption was measured in EY262-cre positive (D1-cre) and EY262-cre negative littermate controls. Both genotypes consumed similar amounts of alcohol (Fig. S2G), ruling out possible off-target effects of Cre expression in D1R-positive cells. Body weight and water consumption were similar for both genotypes, suggesting the differences seen in D1-Lrrk2-KO mice are not due to altered liquid consumption (Fig. S2H) or differences in body weight (Fig. S2I–K)

### Overall unchanged dopamine-related behaviors in mice with *Lrrk2* deletion in D1-MSN

Striatal D1R are involved in the regulation of basic behaviors, such as feeding, motivation, and exploration. Mice from both genotypes showed similar exploratory behaviors in an open-field arena (distance, velocity, % time in center, Fig S3A–C) and similar response to novelty (Fig S3D). Food consumption was measured for regular chow and a high-fat diet (60 kcal% fat, HFD). Body weight and caloric intake was similar between genotypes under both diets (Fig S3F) and mice from both genotypes consumed more calories under HFD than chow (Fig S3F). Body weights were similar at the start of the experiment (Fig S3E) and weight gain was comparable during the different treatments for both genotypes (Fig S3G). Sucrose preference was assessed using a two- bottle choice procedure with 0.5, 1, and 2% sucrose solution. Overall, there was no difference in sucrose preference across genotypes (Fig. S3H). Preference for the non-caloric sweetener sucralose was also similar in both genotypes (Fig S3I).

### Enhanced alcohol response is selective for mice lacking *Lrrk2* in D1R expressing neurons

**Fig. 4:**
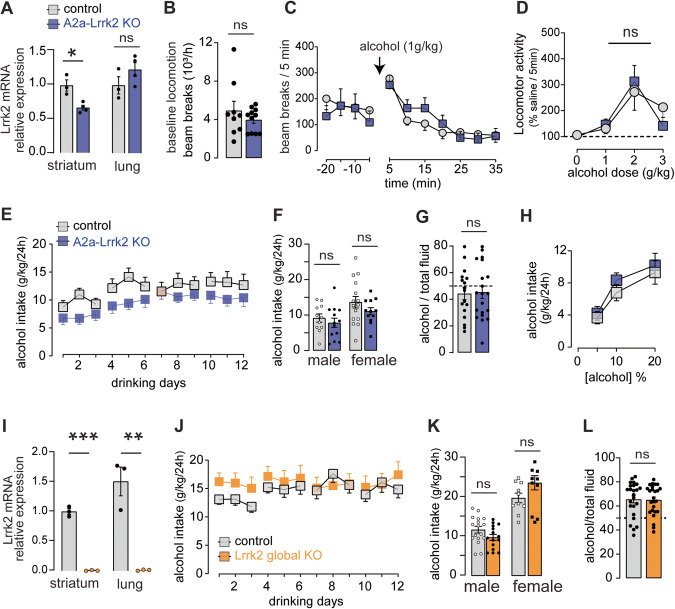
Selectivity of the *Lrrk2* effect on alcohol drinking. **A** Levels of Lrrk2 mRNA expression in the striatum (*t*_(5)_ = 4, *P* < 0.05) and lung tissue (*t*_(5)_ = 1.4, *P* = 0.2) of A2a-Lrrk2-KO mice (blue) and littermate controls (gray). Basal locomotion measured during habituation session (**B**; *t*_(18)_ = 0.97, *P* = 0.34) and systemic administration of alcohol (**C**). **D** Dose-dependence of the locomotor response induced by systemic administration of alcohol (REML: no genotype effect: *F*_(1,31)_ = 0.02, *P* = 0.9). **E** Mean alcohol intake (g/kg/24 h) during intermittent two-bottle-choice sessions (*F*_(1,49)_ = 2.6, *P* = 0.11). **F** Sex differences in the overall alcohol intake. **G** Alcohol preference (*t*_(35)_ = 0.17; *P* = 0.86). **H** Alcohol intake as a function of the alcohol concentration (no genotype effect: *F*_(1,31) _= 0.45, *P* = 0.5). **I** Levels of *Lrrk2* mRNA expression in the striatum and lung tissue of Global-Lrrk2-KO mice (orange) and littermate controls (gray) (*t*_(4) _= 20, *P* < 0.0001; *t*_(4)_ = 6.2, *P* < 0.005). **J** Mean alcohol intake (g/kg/24 h) during intermittent two-bottle-choice sessions (no genotype effect: REML, *F*_(1,52)_ = 0.41, *P* = 0.52). **K** Sex differences in the overall alcohol intake. **L** Alcohol preference (*t*_(47)_ = 0.14, *P* = 0.9). For all panels, bars represent mean ± S.E.M and symbols represent values from individual mice. (*) denotes *P* < 0.05; (**) denotes *P* < 0.01; (***) denotes *P* < 0.001.

To test whether changes in alcohol-related behaviors were contingent on the deletion of the *Lrrk2* gene specifically from D1R expressing neurons, we tested alcohol-induced stimulation and voluntary alcohol consumption in mice lacking the *Lrrk2* gene in D2-MSN. Lrrk2^loxP/loxP^ mice were crossed with Adora2a-cre mice, which express Cre in D2-MSNs within the striatum [35–37], and knockdown efficiency was measured using qPCR (Fig. 4A). Basal locomotion during saline days was similar between genotypes (Fig. 4B). Alcohol produced a transient dose-dependent increase in locomotion that was similar in both genotypes (Fig. 4C, D). No difference on voluntary alcohol drinking (Fig. 4E, F) and preference (Fig. 4G, H) were found (Fig. 4H). Last, we assessed the effect of global deletion of the *Lrrk2* gene on alcohol consumption. qPCR analysis confirmed the global deletion of the *Lrrk2* gene (Fig. 4I). Global-Lrrk2-KO mice consumed similar amounts of alcohol than controls (Fig. 4J, K) and showed similar alcohol preference (Fig. 4L). Water consumption (Fig. S3G) and body weight were also similar across genotypes (Fig. S3H).

### Alcohol lowers LRRK2 kinase activity in the dorsal striatum

We tested whether alcohol drinking modulates LRRK2 kinase activity in the striatum of C57BL6/J wild-type mice. LRRK2 activity was assessed via quantification of the phosphorylation levels of LRRK2 at residue S935, a phospho-site that is decreased by LRRK2 kinase inhibitors, and via phosphorylation levels of the LRRK2 substrate Rab10 [38, 39]. A single session of alcohol consumption (Fig. S4A) promoted a decrease in the levels of pS935-LRRK2 in DMS and DLS (Fig. S4B) and a specific decrease in the levels of pT73-Rab10 in the DMS (Fig. S4C) 48 h after the drinking session. The reduction of pT73-Rab10 was correlated with the amount of alcohol consumed (Fig. S4D). Total levels of LRRK2 and Rab10 were not affected by alcohol drinking. However, there was a regional difference in the levels of LRRK2, which were higher in the DLS compared to the DMS (Fig. S4F).

Passive administration of alcohol (2 g/kg, i.p.) produced a significant reduction in pT73- Rab10 levels which peaked after 12 h of administration and was recovered by 48 h (Fig. S4I). No significant change in pS935-LRRK2 levels was observed, but there was a trend of reduction (Fig. S4J).

### Enhanced likelihood of heavy and punishment-resistant alcohol drinking

**Fig. 5:**
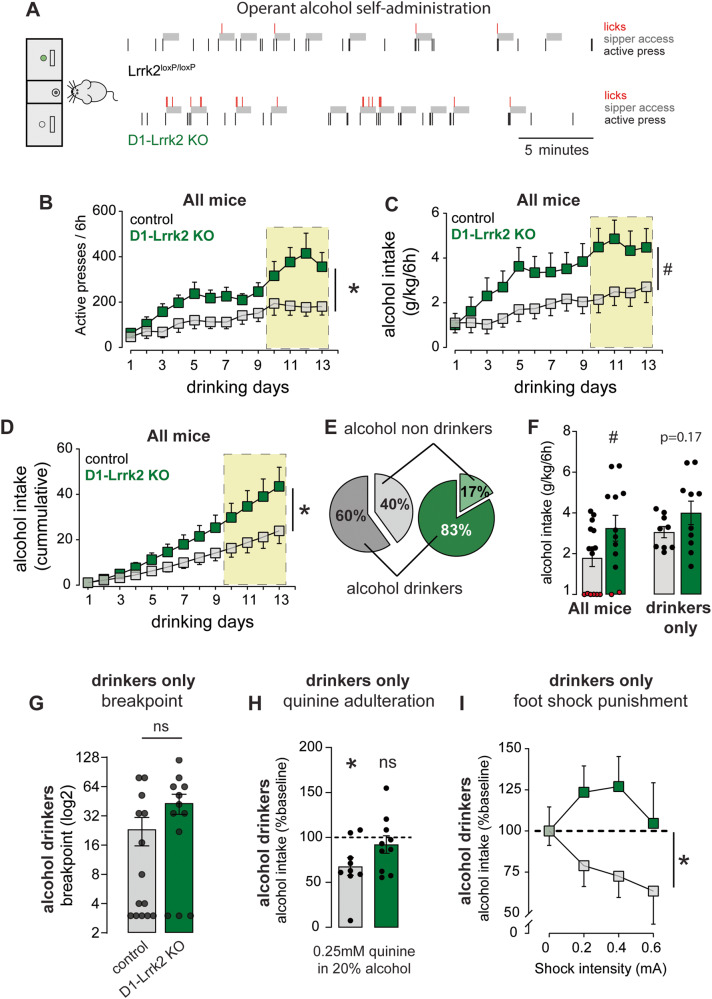
*Lrrk2* expression in D1R-expressing neurons promotes resilience to punishment-resistant alcohol drinking in mice. **A** Representative raster plot of the first 30 min of a training session at FR3 for a control mouse (top) and a D1-Lrrk2-KO mouse (bottom). Three responses in the active lever (black bar) lead to 60 s of access to a sipper tube containing 20% alcohol (gray shaded area). Licks responses are shown in red. **B** Mean rate of active lever pressing during the operant drinking sessions. Shaded yellow area represents FR3 sessions (genotype: *F*_(1,25)_ = 5.7; session: *F*_(3.5,88)_ = 15 and interaction: *F*_(12,300)_ = 2.6, *P*s < 0.05). **C** Average daily alcohol intake (g/kg/6 h) (*F*_(1,25)_ = 4, *P* = 0.056). **D** Average cumulative daily alcohol intake (g/kg/6 h) (interaction: *F*_(12,300)_ = 4.22; *P* < 0.0001). **E** Proportion of alcohol drinkers and non-drinkers. **F** Average of alcohol consumption (g/kg/6 h) among all mice (*t*_(25)_ = 2, *P* = 0.056) and among alcohol drinkers only (*t*_(17)_ = 1.4, *P* = 0.17); the red circles represent the individual average consumption of non-drinkers. **G** Breakpoint during progressive ratio session (Mann–Whitney *U*(58); *P* = 0.18). Percent of change in alcohol intake during a quinine adulteration session (**H**; one-sample *t* test, D1-Lrrk2-KO: *t*_(8)_ = 3.3, *P* < 0.05; control: *t*_(9) _= 0.8, *P* = 0.44) and during foot shock sessions (**I**; genotype effect: *F*_(1,17)_ = 5.6, *P* < 0.05) compared to baseline consumption. For all panels, data from Lrrk2^loxP/loxP^ is shown in gray and from D1-Lrrk2-KO in green; bars represent mean ± S.E.M and symbols represent values from individual mice. * denotes *P* < 0.05; (#) denotes *P* = 0.056.

We used an operant alcohol self-administration paradigm (SA) to assess motivation and resistance to punishment, two core manifestations of AUD [40, 41] (Fig. S5A). Mice were initially exposed to a drinking in the dark (DID) procedure for 4 weeks. Average alcohol intake during DID was similar between genotypes (3.9 ± 0.2 g/kg/4 h vs 4 ± 0.3 g/kg/4 h; *t(*26) = 0.23, *P* > 0.05). Mice were then trained to press an active lever to gain access to a sipper tube containing 20% alcohol solution for 60 s. D1-Lrrk2-KO mice showed a higher rate of responding on the active lever and earned longer access time to alcohol compared to controls (Fig. 5A, B). D1-Lrrk2-KO mice showed a strong trend towards larger alcohol consumption than controls (Fig. 5C). Overall, the cumulative alcohol consumption of D1-Lrrk2-KO mice was 1.8-fold significantly higher than controls (Fig. 5D). The rate of licking in the sipper spout was similar across genotypes (Fig. S5C). Notably, a higher percentage of D1-Lrrk2-KO mice successfully acquired the task compared to controls (Fig. 5E, F). Within the mice that acquired the task (“drinkers”), D1-Lrrk2-KO displayed a noticeable trend towards higher alcohol consumption (Fig. 5F) and showed higher rate of acquisition than controls (Fig S5D).

Only mice that reached criteria for acquisition were tested on the other aspects of the operant task to assess motivation and the response to punishment. Breakpoint response was measured in a single progressive ratio session and was similar in both genotypes (Fig. 5G). Drinking despite negative consequences was assessed by measuring the degree of suppression of alcohol drinking during quinine adulteration or foot-shock sessions. During quinine adulteration, control mice showed a significant reduction in drinking while D1-Lrrk2-KO mice maintained an average intake similar to baseline levels (Fig. 5H; S5E). Neither genotype showed a significant change in lever press rate (Fig. S5E). Suppression of alcohol drinking by quinine was also measured using a non-operant paradigm in a separate cohort of mice. Both genotypes reduced drinking when alcohol was adulterated with 0.5 mM quinine. However, D1-Lrrk2-KO mice showed only a small reduction in drinking when alcohol was adulterated with 0.25 mM quinine (Fig. S5F). No genotypic differences in taste sensitivity to quinine were found (Fig. S5G).

During foot shock sessions, D1-Lrrk2-KO mice maintained high levels of alcohol drinking at all shock intensities whereas controls substantially reduced alcohol consumption (Fig. 5I, S5J). D1-Lrrk2-KO mice, but not controls, showed a reduction in the number of lever presses that was dependent on shock intensity (Fig. S5H). Despite the reduction in the number of lever presses, D1- Lrrk2-KO mice earned slightly more rewards than controls during foot shock sessions and received slightly higher total number of shocks throughout sessions, although these differences were not statistically significant (Fig. S5I, K). Pain threshold and shock threshold sensitivity were similar between D1-Lrrk2-KO and control mice (Fig. S5L, M).

We also assessed the effect of quinine adulteration and foot shock on sucrose SA behavior. Both genotypes self-administered similar amounts of sucrose at all concentrations tested (Fig. S6A) and showed similar breakpoints responses (Fig. S6B). Sucrose drinking was robustly suppressed during quinine adulteration and foot shock sessions in both genotypes (Fig. S6C).

## Discussion

This study uncovers a novel role for the Parkinson’s-related gene *Lrrk2* in regulating D1R function and alcohol drinking. Loss of LRRK2 function in neurons expressing D1R potentiates D1R signaling and function, alcohol reinforcement, and punishment-resistant alcohol drinking.

LRRK2 is a complex protein involved in regulating various neuronal functions, such as vesicular trafficking, autophagy, and cytoskeleton dynamics [7, 42–45]. Mutations in human *LRRK2* are commonly linked to Parkinson’s disease, indicating its important role in regulating basal ganglia function [9]. We found that constitutive deletion of *Lrrk2* from D1R expression neurons promotes D1R function at the cellular, synaptic, and behavioral level. This enhanced D1R activity in D1- Lrrk2-KO mice suggests a sensitized-like state of D1 receptors, similar to sensitization observed following dopamine depletion [46, 47]. For example, we found that low doses SKF81297 increased c- Fos expression in D1-MSNs only in D1-Lrrk2-KO mice. This finding resembles previous published observations with low dose agonist following sensitization of dopamine receptors in wild-type mice [48]. Evaluation of cFos expression in other brain regions relevant to AUD and where D1R and *Lrrk2* might be co-expressed would be a valuable exploration for future studies.

The PKA inhibitor PKI revealed a role for PKA in regulating D1-MSM excitability and D1R signaling at the soma of D1-MSNs. The exact molecular mechanisms involved still need to be fully elucidated; although LRRK2 modulation of PKA localization and D1R surface expression are very likely involved, in agreement with previous published reports [12–14].

The cellular and behavioral response to alcohol was enhanced in D1-Lrrk2-KO mice. Acute alcohol application to the brain slices containing the striatum revealed a dose dependent effect on the firing of D1-MSNs, which was stronger in D1-Lrrk2-KO compared to controls at high alcohol concentrations. Alcohol modulates the activity of voltage-gated ion channels in MSNs to regulate intrinsic excitability [49–52]. Future studies are needed to identify the specific ion channels and molecular mechanisms by which LRRK2 regulates neuronal intrinsic excitability, most likely via PKA. Postnatal deletion of the *Lrrk2* gene in fully developed brains did not replicate the effect observed in D1-Lrrk2-KO mice, suggesting that loss of LRRK2 function early in development or cell-specificity in the deletion is essential for D1R potentiation. Previous research has already demonstrated the involvement of LRRK2 in synapse formation in the striatum, as evidenced by impaired synaptic plasticity in young Lrrk2-KO and KI mice, but not in adult mice. [7, 14, 53] These findings are in agreement with our results and suggest that loss of *Lrrk2* delays synapse formation in the striatum. We propose that this role of *Lrrk2* in striatal synapse formation might explain the lack of a behavioral phenotype when *Lrrk2* deletion was achieved postnatally in our study.

Alcohol-related behaviors that are dependent on D1R activity, such as alcohol stimulation, sensitization, and intake, were enhanced in mice with *Lrrk2* deletion from D1R-expressing neurons, even though baseline dopamine-related behaviors were not affected overall. Alcohol- induced sedation was not affected, consistent with previous findings suggesting that alcohol sedation is unlikely to be dependent on dopamine receptors [54–56]. These findings suggest a potential link between the deletion of the *Lrrk2* gene and the promotion of alcohol-related behaviors via an enhancement of D1R signaling. However, it is essential to emphasize that this association requires further investigation and validation in order to establish causality. With regard to drinking behavior, D1-MSN activity is known to modulate alcohol rewarding properties and drinking [57, 58]. D1-Lrrk2-KO mice showed higher alcohol preference and consumption than controls, reaching blood alcohol levels above the intoxication level of 80 mg/dl during intermittent alcohol access, suggesting that alcohol was consumed because of its pharmacological effects. We observed sex differences in alcohol drinking, corroborating findings from existing literature. Nonetheless, it is important to acknowledge that the other behavioral tests used here were underpowered to detect such differences adequately. D1-Lrrk2-KO mice displayed higher alcohol consumption and continued drinking despite negative consequences, as measured during punishment sessions using both quinine-adulteration and foot-shock. Interestingly, despite the reduction in the number of lever presses, D1-Lrrk2-KO mice earned more rewards than the controls during the foot-shock paired session. This result could indicate a deficit in associative learning that is specific for positive punishments, or the development of a different strategy during the foot-shock paired sessions. Pain and taste sensitivity thresholds were not altered in the D1-Lrrk2-KO, ruling out their contribution to punishment- resistant alcohol drinking. Deletion of *Lrrk2* did not affect motivation to drink or seek alcohol, as there was no difference in breakpoint response. This dissociation between motivational salience and response to punishment is consistent with pre-clinical models of cocaine use disorder [59, 60].

It is important to note that our results only offer correlative evidence of enhanced D1R activity and higher alcohol drinking in Lrrk2-KO mice. Further research is needed to establish a causal link between D1R activity and alcohol consumption in these mice. Additionally, while our study focused on postsynaptic dopamine dynamics in the striatum, it is reasonable to consider that behavioral changes may also involve pre- and postsynaptic mechanisms. The complex nature of the striatal microcircuitry suggests that enhanced D1R activity could indirectly impact the physiology of D2-MSNs, contributing to the observed behavioral changes. Exploring the role of D2R and the interplay between pre- and postsynaptic mechanisms presents an intriguing avenue for future research.

Increased alcohol consumption in D1-Lrrk2-KO mice is not due to generalized increase in motivation for natural rewards or taste sensitivity, as demonstrated by the lack of differences in sucrose preference, non-caloric sweetener preference, regular food, or HFD consumption. In our previous study, we found a positive correlation between *Lrrk2* mRNA levels and drinking despite taste adulteration in mice [10], seemingly contradicting our current findings. We hypothesized that the increase in *Lrrk2* mRNA is a homeostatic response to the inhibition of LRRK2 activity induced by alcohol, and this response is stronger in vulnerable subjects, either because the alcohol suppression is stronger or the baseline levels of LRRK2 activity are lower. Notably, the changes in alcohol-related behaviors were specific to mice lacking *Lrrk2* in D1R expressing neurons, including D1-MSNs, and not in those lacking the gene in D2-MSNs or in global Lrrk2-KO mice. This cell-specificity aligns with previous findings related to DARPP-32, where deletion in both cell types resulted in no net change due to the balance between D1-MSNs and D2-MSNs [61, 62]. Deletion of *Lrrk2* in D2-MSNs seems sufficient to prevent the potentiation of D1R function when *Lrrk2* is deleted in both cell types, suggesting that the balance of activity between these two pathways collectively regulates alcohol drinking behavior. Another recent study supports this concept of balance, finding that rats with uncontrolled alcohol-seeking have higher levels of Drd1a mRNA and lower levels of Drd2 mRNA in the striatum [63]. However, it is worth noting that *Lrrk2* is expressed in various brain regions and cell types, and the lack of phenotype in the Global-Lrrk2- KO mice may involve a more complex phenomenon. Further investigations are required to delve deeper into this proposed mechanism and elucidate the intricate dynamics involved in the regulation of alcohol drinking behavior in the absence of *Lrrk2*.

The mechanisms driving vulnerability to alcohol abuse likely extend beyond the striatum. Our KO strategy deleted *Lrrk2* not only in the striatum but also in all D1R-expressing neurons, including those in different cortical areas, amygdala, and hippocampus. It is also possible that developmental alterations play a role due to constitutive deletion of *Lrrk2*. Further studies are needed to investigate the consequences of postnatal disruption of the *Lrrk2* gene or pharmacological inhibition of LRRK2 kinase activity to assess its developmental role. We found that a single episode of heavy alcohol drinking decreased LRRK2 kinase activity, bidirectional regulation of alcohol drinking by LRRK2. We speculate that vulnerability to AUD arises from the interaction between developmental changes in LRRK2 function and acute modulation of LRRK2 by alcohol. For instance, low LRRK2 activity in D1-MSNs could prime D1Rs to sensitization upon alcohol exposure, and acute decrease in LRRK2 function by alcohol could further promote D1R hypersensitivity. These findings reveal an important developmental role of *Lrrk2* in the striatal function and suggest possible synergism between genetic and developmental factors in the development of complex behaviors and disorders, including vulnerability to AUD.

In conclusion, we propose that LRRK2 negatively regulates D1R function in D1-MSNs, limiting receptor signaling and the actions of alcohol that are mediated via D1Rs. LRRK2 activity thus limits the reinforcing properties of alcohol and is expected to promote resilience to heavy alcohol drinking, especially when paired with adverse consequences. Pharmacological manipulations of LRRK2 might hold therapeutic promise in reducing uncontrolled heavy drinking of alcohol.

### Supplementary information

Supplementary Files

Supplemental Table 1

## Data Availability

All data needed to evaluate the conclusions in the paper are present in the paper and/or the Supplementary Materials.
