# Supplementary material for: Leucine-rich repeat kinase 2 limits dopamine D1 receptor signaling in striatum and biases against heavy persistent alcohol drinking
Source: Neuropsychopharmacology. 2023 Sep 8;49(5):824–36. doi: 10.1038/s41386-023-01731-z (PMC10948780; doi:10.1038/s41386-023-01731-z)
Supplement: Supplementary file 1 — Supplementary Files [file 41386_2023_1731_MOESM1_ESM.pdf]

“Leucine-Rich Repeat Kinase 2 limits dopamine D1 receptor signaling in striatum and biases against heavy persistent alcohol drinking”

da Silva et al.

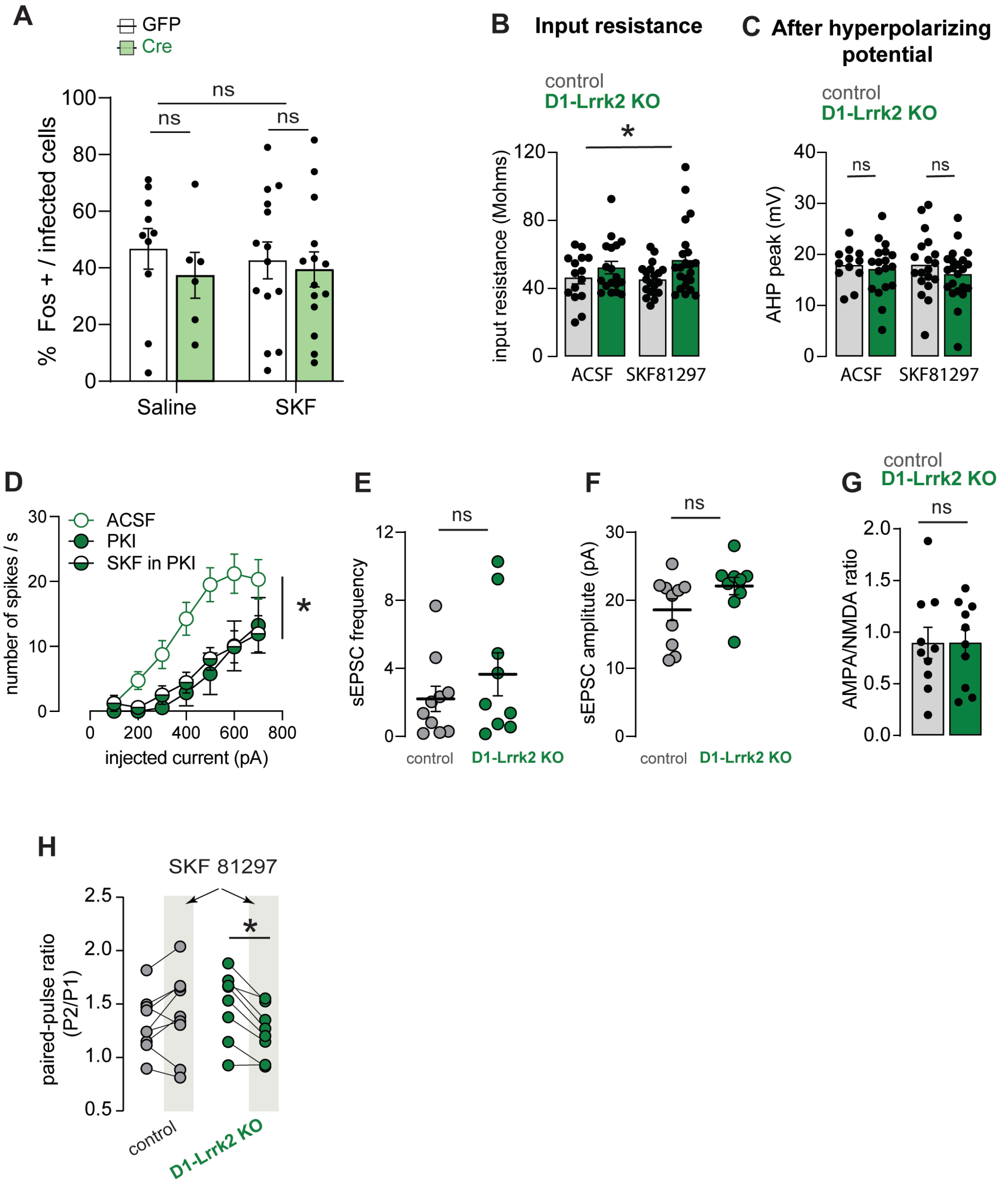

Fig S1: c-Fos quantification and assessment of D1R, Glutamatergic, and GABAergic function in D1-Lrrk2-KO mice.

A, Quantification of c-Fos positive cells in the striatum of Lrrk2$^{floxP/floxP}$ mice injected with either Cre-eGFP or GFP after systemic administration of saline or SKF81297 (2 mg/kg; no virus, treatment or interaction effect: $F_{(1,40)}$=0.17, 0.017, 0.72, *Ps*>0.4). **B**, Input resistance recorded in baseline conditions and in the presence of SFK81297 (main effect of treatment:$F_{(1,71)}$=5.6, *P*<0.05). **C,** After hyperpolarizing current measured in baseline conditions and in the presence of SKF81297 (no genotype effect: $F_{(1,67)}$=1.0; no

interaction: $F_{(1,67)}$=0.16; *Ps*>0.05). **D**, Input-output curve of firing rate from D1-MSNs in response to current steps of increasing amplitude after incubation of slices with SFK81297 and PKA inhibitor PKI (PKI effect: $F_{(1,26)}$=8.7, *P*<0.01; interaction: $F_{(6,156)}$=2.2, *P*<0.05; n=9 cells, 3 mice) E-F, spontaneous postsynaptic current frequency (E, $t_{(17)}$=1, *P*=0.33) and amplitude (F, $t_{(17)}$=1.7, *P*=0.11) in D1-Lrrk2 KO and control mice. **G**, Average AMPAR/NMDAR ratio $t_{(18)}$=0.01, *P*=0.99, n=10/10 cells, 4/3 mice. **H**, Paired pulse ratio of the synaptic responses before and after (shaded area) bath application of D1-like agonist (interaction: $F_{(1,15)}$=16, *P*<0.001; Sidak's test: control, *P*=0.26; D1-Lrrk2-KO, *P*<0.005). For all panels, bars represent mean ± S.E.M and symbols represent values from individual mice. * denotes *P* < 0.05;

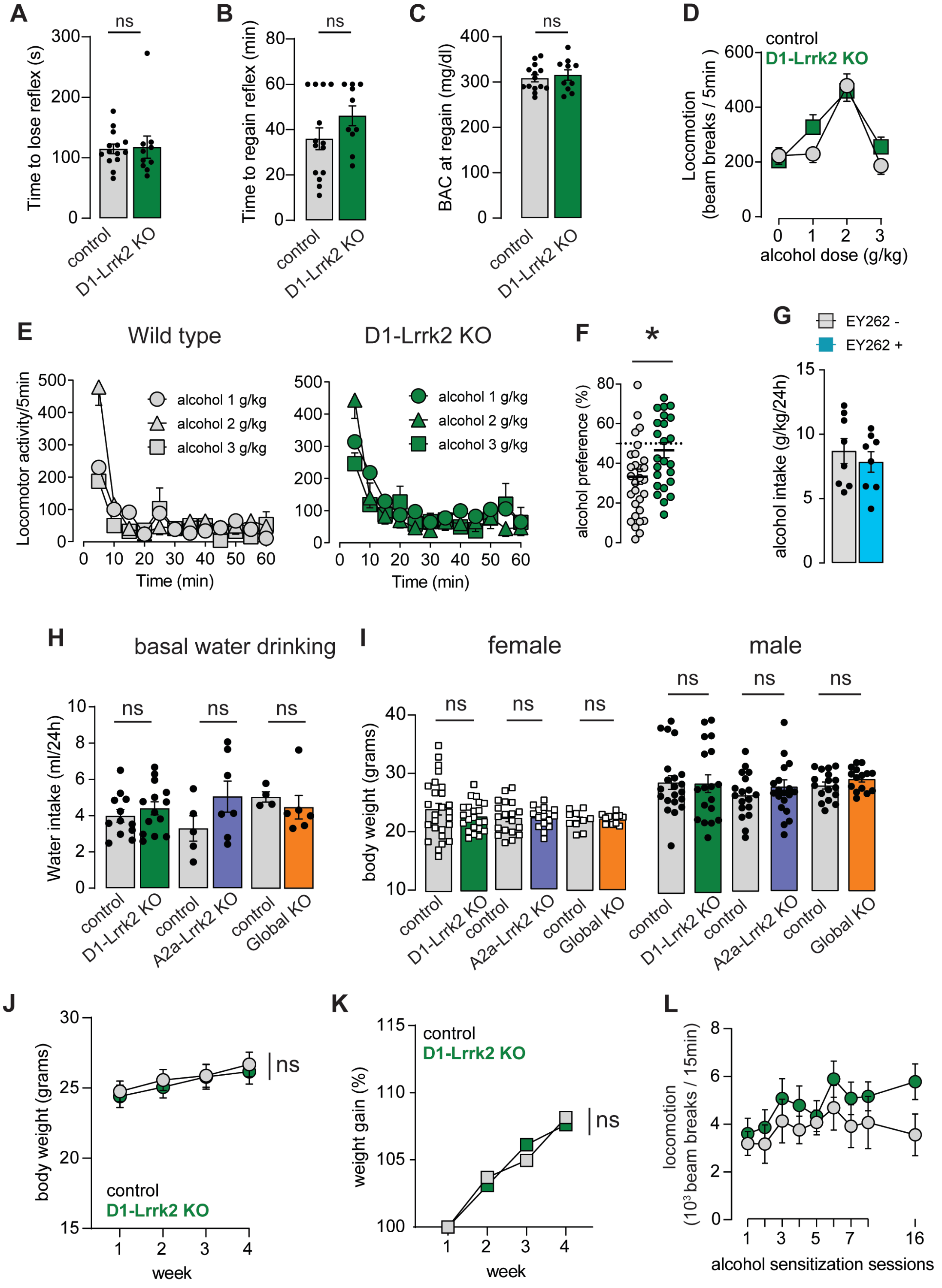

Fig S2: LORR and consummatory behaviors in cell-specific Lrrk2-KO mice.
A, Latency to lose the righting reflex ($t_{(22)}$=0.16, *P*=0.87). **B**, Time to regain the righting reflex ($t_{(22)}$=1.5, *P*=0.15). **C**, BAC at the time of regaining the righting reflex ($t_{(22)}$=0.56, *P*=0.6). **D**, Raw data of dose-dependent locomotor response induced by systemic administration of alcohol. **E**, Time course of the effects of different doses of alcohol on locomotion. **F,** Mean alcohol preference during 2-bottle-choice sessions for

D1-Lrrk2 KO (green) and littermate controls (gray) ($t_{(53)}$=2.4, P<0.05). **G,** Mean alcohol intake (g/kg/24h) during 2-bottle-choice sessions for EY262 (blue) and littermate controls (gray) ($t_{(14)}$=0.7 P=0.51). **H,** Bar graphs showing basal water drinking (ml/24h) for D1-Lrrk2-KO (green; $t_{(24)}$=0.83), A2a-Lrrk2-KO (blue; $t_{(10)}$=1.5), Global-Lrrk2-KO (orange; $t_{(8)}$=0.7) and their respective littermate controls (gray). **I,** Bar graphs showing average body weight for females (left) and males (right) for D1-Lrrk2-KO (green; $t_{(37)}$=0.17; $t_{(46)}$=1.1), A2a-Lrrk2-KO (blue; $t_{(36)}$=0.9; $t_{(33)}$=0), Lrrk2-Global-KO (orange; $t_{(30)}$=1.4; $t_{(20)}$=0.2), and their respective littermate controls. **J-K,** Raw (J) and percentage (K) of weight gain for D1-Lrrk2-KO and controls during 4 weeks of drinking procedure. **L**, Locomotor response to eight consecutive daily injections of alcohol followed by a challenge injection after eight days of incubation. For all panels, data from $Lrrk2^{loxP/loxP}$ is shown in gray and from D1-Lrrk2-KO in green; bars represent mean ± S.E.M and symbols represent values from individual mice.

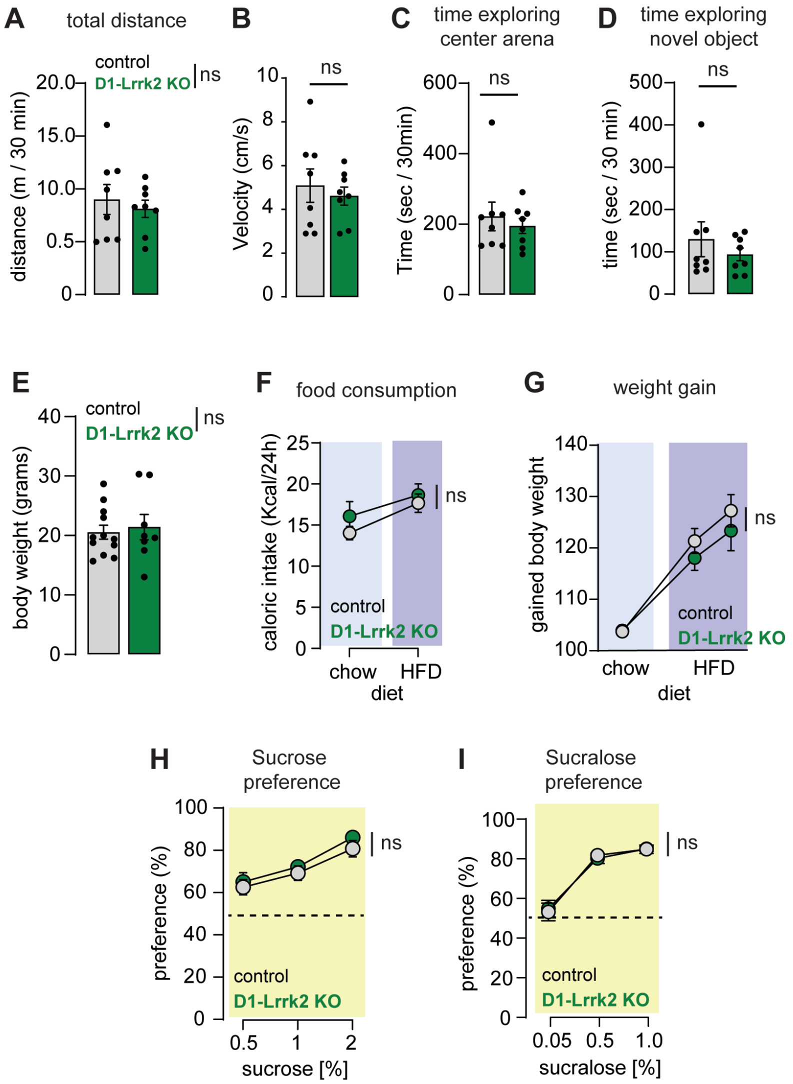

Fig S3: Exploration and caloric intake are largely unchanged following selective deletion of *Lrrk2* in D1-MSN.

A-D, Distance travelled ($t_{(14)}$=0.53, P=0.6), velocity($t_{(14)}$=0.55, P=0.59), time in center ($t_{(14)}$=0.6, P=0.56) and novel object exploration ($t_{(14)}$=0.8, P=0.42) assessed in an open field arena. **E**, average body weight for age-matched mice of each genotype ($t_{(18)}$=0.4, P=0.7). **F**, Caloric intake in Kcal/day when given unrestricted access to normal chow diet and high fat diet (HFD) for littermate control (gray) and D1-Lrrk2-KO mice (green) (genotype: $F_{(1,18)}$=1.3, P=0.26; diet: $F_{(1,18)}$=6.8, P<0.05). **G**, change in body weight during access to regular chow diet or HFD diet (no genotype: $F_{(1,18)}$=0.6, P=0.44; no interaction: $F_{(2,36)}$=0.7, P=0.7). **H**. Sucrose preference during 0.5%, 1% and 2% sucrose solution access using a two-bottle choice procedure (REML, no genotype effect: $F_{(1,42)}$=1.7, P=0.2; dose effect: $F_{(2,62)}$=18, P<0.0001). **I**, Sucralose preference using two-bottle choice procedure (concentration effect: $F_{(1.35,44.8)}$=68, P<0.0001; no genotype effect: $F_{(1,33)}$=0.001, P=0.97). For all panels, data from $Lrrk2^{loxP/loxP}$ is shown in gray and from D1-Lrrk2-KO in green.

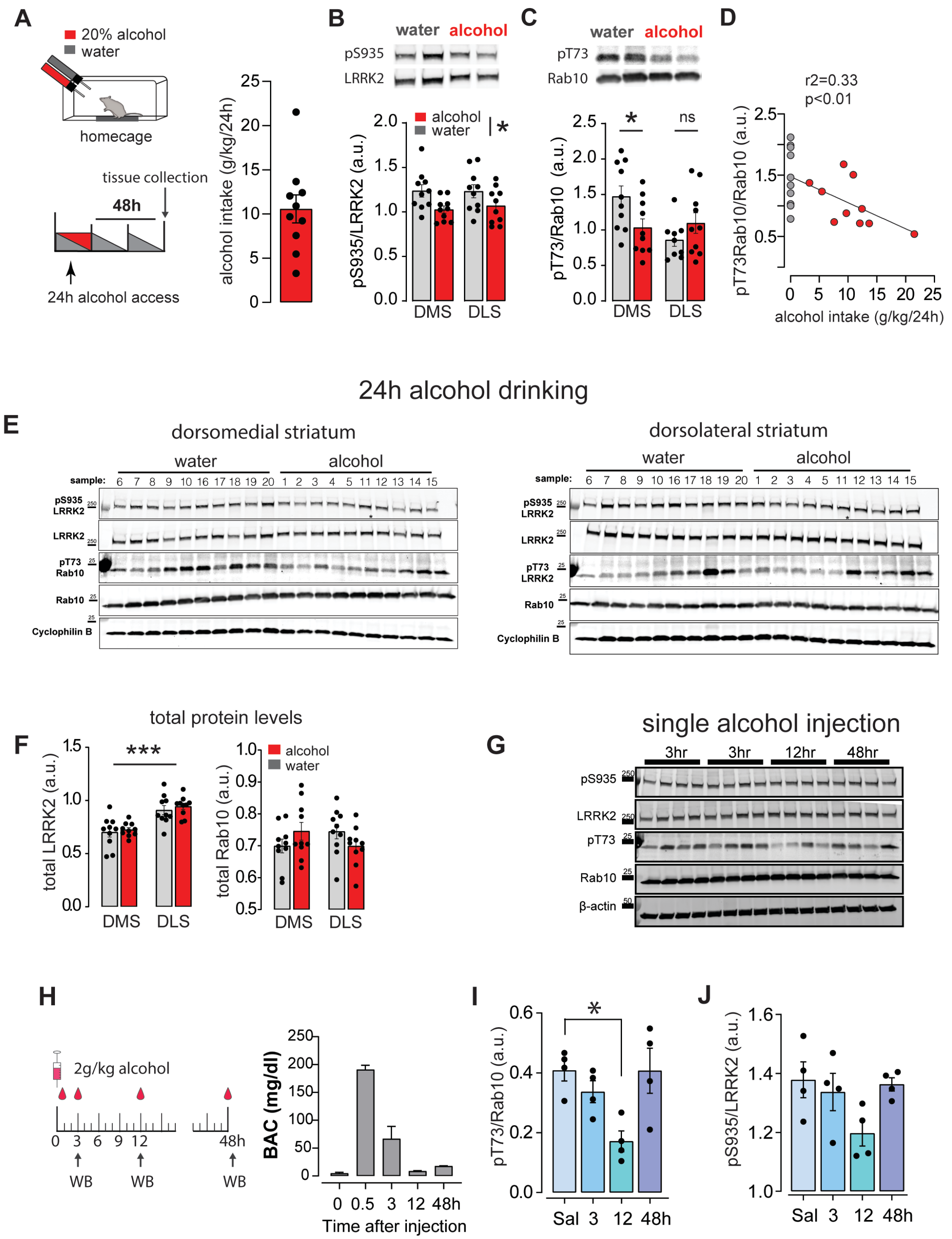

Fig S4: Alcohol modulates LRRK2 activity in mouse striatum.

A, *Top*, schematic diagram of the two-bottle-choice paradigm used to measure volitional alcohol drinking over 24h. *Bottom*, time course of the experiment outlines 24h alcohol drinking and brain tissue collection for western blots (WB) 48h after the single drinking session. R*ight*, Average alcohol consumed over 24h. **B-C,** *Top*, Images of Western Blots for pS935 LRRK2 and total LRRK2 and pT73-Rab10 and total Rab10

in samples from DMS and DLS of water and alcohol drinking mice. *Bottom*, bar graphs showing the ratio of labeling density for the phosphorylated forms of LRRK2 (alcohol effect: $F_{(1,18)}=5.3$, $P<0.05$; no interaction: $F_{(1,18)}=0.4$, $P=0.53$) and Rab10 (interaction: $F_{(1,17)}=14.4$ $P<0.005$; DMS Sidak's test $P<0.05$) over the total protein levels. **D,** pT73-Rab10 as a function of the amount of alcohol consumed over a single 24h drinking session ($r^2 = 0.33$, $P<0.01$). **E,** Western Blots for pS935 LRRK2, LRRK2, pT73 RAB10, RAB10, and Cyclophilin B in the DMS (*left*) and DLS (*right*) for alcohol and water drinking mice. **F,** Bar graphs showing labeling density for total LRRK2 (*left;* no genotype effect: $F_{(1,18)}=0.56$; region effect: $F_{(1,18)}=42$, P<0.0001) and total Rab10 (*right; no genotype effect* $F_{(1,18)}=0$, *P=0.99) i*n the DMS and DLS of water (gray) and alcohol (red) drinking mice. **G,** Western Blots for pS935 LRRK2, LRRK2, pT73 RAB10, RAB10, and ß-actin in the DMS of mice injected with saline or 2g/kg alcohol. **H,** *Left*, Schematic diagram of systemic injections of 2g/kg alcohol and time points of tissue collections for Western blot. *Right*, Blood alcohol levels in different time points after systemic injection of 2g/kg alcohol. **I-J,** Time-dependent changes in pS935 LRRK2 (I; effect of time: $F_{(3,12)}=2.7$, $P=0.09$) and pT73 RAB10 (J; time effect: $F_{(3,12)}=5.2$, $P<0.05$; Tukey's test: Saline *vs* 12h, $P<0.05$) levels after systemic injection of 2g/kg alcohol. For all panels, bars represent mean ± S.E.M and symbols represent values from individual mice. * denotes $P < 0.05$, *** denotes $P < 0.0001$

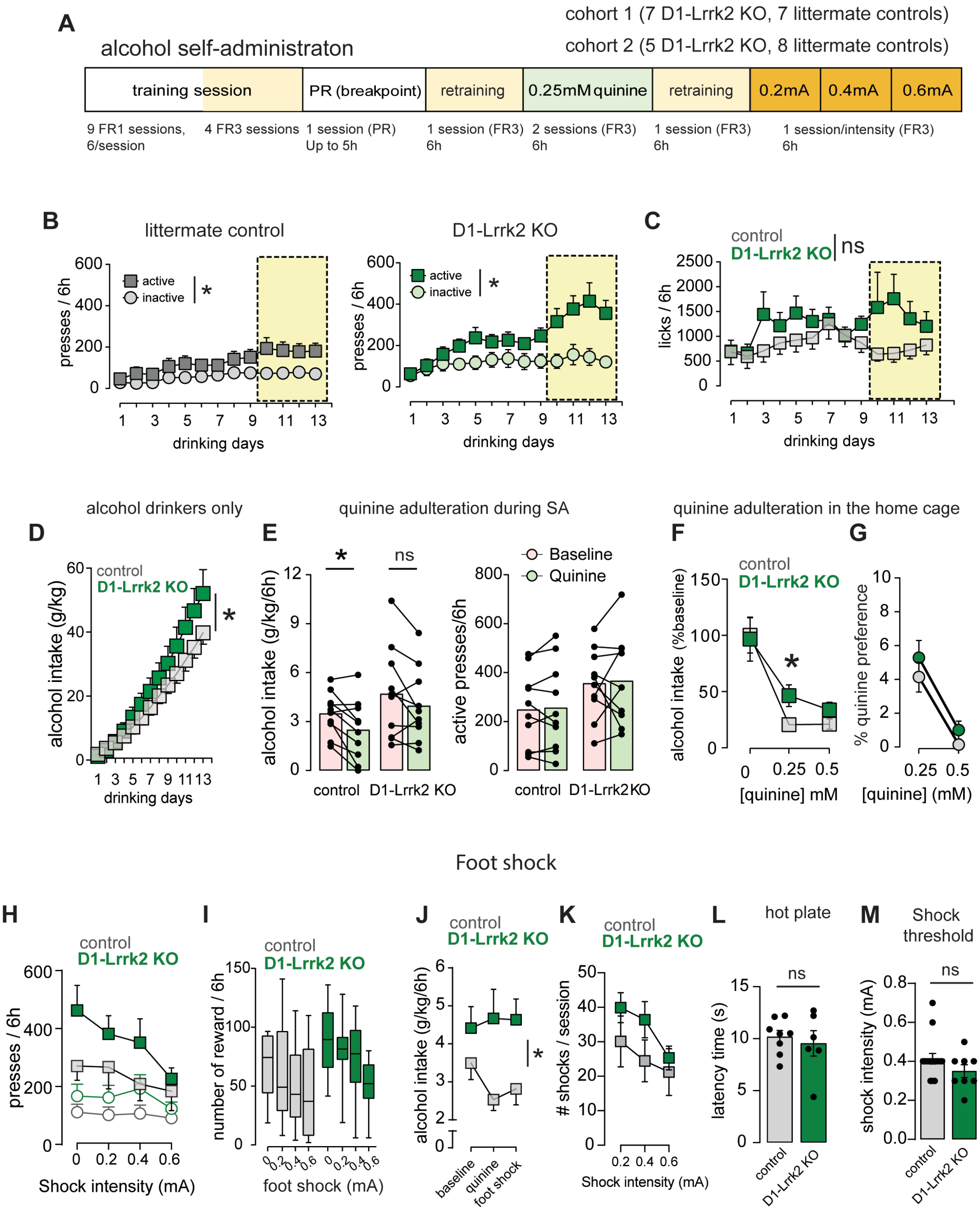

Fig S5: Operant alcohol self-administration in D1-Lrrk2-KO mice.

A, Detailed schematic diagram of the operant training schedule. **B,** Mean number of active and inactive lever presses (lever response: $F_{(1,14)}$=16, P<0.005). **C,** Mean number of licks (no genotype effect: $F_{(1,25)}$=1.38, P=0.21). **D**, Average cumulative daily alcohol intake (g/kg/6h) among the population of alcohol drinking mice (interaction: $F_{(12,204)}$=2.3; P<0.05). **E,** Average alcohol intake (main effect of dose $F_{(1,17)}$=11, P<0.005) and active lever presses (right; no effect of dose $F_{(1,17)}$=0.22, P=0.65) during baseline (pink) and quinine adulteration (*green*) sessions. **F,** Non-operant alcohol intake (g/kg/24h) during baseline sessions with access to 20% alcohol solution (0) and during quinine-adulteration sessions(genotype effect: $F_{(1,19)}$=5.0, P<0.05; Sidak's test, P<0.05). **G,** Quinine preference (taste aversion) tested using two bottle choice for tap water or quinine solution (no genotype effect $F_{(1,12)}$=1.4, P=0.25). **H,** Mean number of lever presses during foot shock sessions (main effect of shock intensity: $F_{(1.5,38.2)}$=2.6, P=0.1; interaction: $F_{(2,50)}$=2.3, P=0.1). **I**, Average number of earned rewards during baseline and foot shock sessions (effect of shock intensity $F_{(2.4,41)}$=10.6, P<0.0001). **J**, Alcohol intake (g/kg) during baseline, quinine adulteration and foot shock SA sessions (main effect of genotype $F_{(1,17)}$=6.2, P<0.05). **K**, Average number of shocks received during foot shock punishment sessions. **L**, Average withdrawal latency in the hot plate test (; $t_{(12)}$=0.51, P=0.6). **M**, Average threshold sensitivity to foot shock (Mann Whitney U=41.5; P=0.18). For all panels, data from Lrrk2$^{loxP/loxP}$ is shown in gray and from D1-Lrrk2-KO in green; bars represent mean ± S.E.M and symbols represent values from individual mice. * denotes P < 0.05

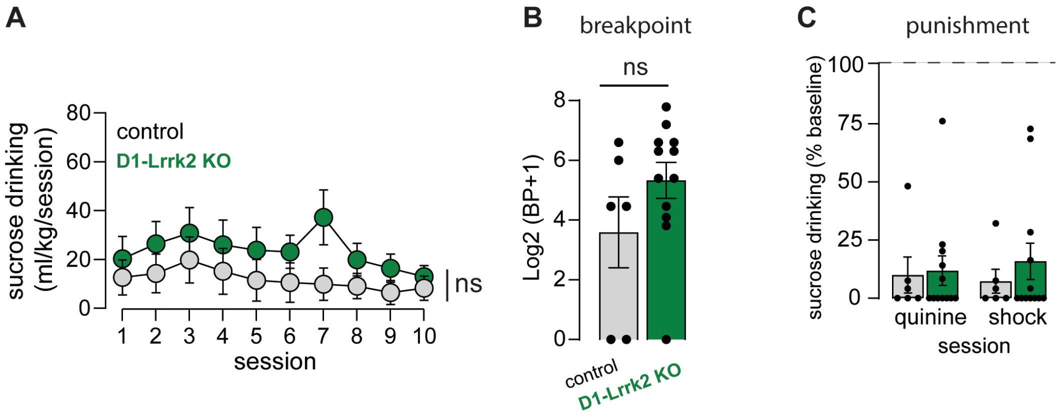

Fig S6: Operant sucrose self-administration in D1-Lrrk2-KO mice.
A, Sucrose consumption during operant self-administration task (no genotype effect: $F_{(1,44)}$=1.4, P=0.24; $t_{(44)}$=1.1, P=0.26). **B**, Breakpoint for sucrose (U(23), P=0.23). **C**, Sucrose drinking during quinine adulteration ($t_{(16)}$=0.17, P=0.86) and foot shock ($t_{(16)}$=0.73, P=0.48) punishment sessions. Data from Lrrk2$^{loxP/loxP}$ is shown in gray and from D1-Lrrk2-KO in green.

“Leucine-Rich Repeat Kinase 2 limits dopamine D1 receptor signaling in striatum and biases against heavy persistent alcohol drinking”
da Silva et al.

Supplementary Material and Methods

Electrophysiology: Slices used for the excitability experiments were incubated in SKF81297 (1 μM), ethanol (50 mM), ethanol (100 mM), and PKI (1 μM) for 20 mins before the start of recordings (maximum time 120 min). tdTomato positive D1-MSN were visualized with a 40x water-immersion objective on an upright fluorescent microscope (BX51WI, Olympus USA). Membrane responses to 1s current injection between 0-700 pA (100 pA increment) were collected using an Axopatch-200B amplifier (Molecular Devices). Current was adjusted to keep Vm at -90 mV. Patch pipettes (2.0-3.5 MΩ) were filled with internal solution containing (in mM): 120 K-$MeSO_4$, 20 KCl, 2 $MgCl_2$, 10 HEPES, 0.2 K-EGTA, 4 Na-ATP, and 0.4 Na-GTP, pH 7.35, 290 mOsm. For whole-cell voltage clamp recordings, neurons were voltage clamped at -60 mV. Paired pulse light (0.2-2 ms) and electrical stimulation (20 Hz every 30 s) were used to trigger GABA-A IPSCs in GABA neurons in SNr. Physiological identification of GABA neurons was based on the rate of spontaneous action potential (>5 Hz) with spike widths <1.2 ms. Internal solution contained (in mM): 57.5 KCl, 57.5 K-methylsulfate, 20 NaCl, 1.5 $MgCl_2$, 5 HEPES, 10 BAPTA, 2 ATP, 0.2 GTP, 10 phosphocreatine, pH 7.35, 290 mOsM. To isolate GABA-A currents, NBQX (5 μM) and CPP (5 μM) were added to aCSF. Series resistance was monitored throughout the experiment (range; 3-15 MΩ). AMPAR/NMDAR ratio was recorded using patch pipette filled with internal solution containing (in mM): 120 $CsMeSO_3$, 10 CsCl, 10 HEPES, 0.2 EGTA, 4 ATP, 0.4 GTP, and 10 phosphocreatine, pH 7.35 (290 mOsM). Evoked EPSCs were generated by electrical stimulation and recorded in presence of gabazine (5 μM). AMPAR current was recorded at -60 mV and NMDAR current was measured at 40 mV in the presence of NBQX (5 μM). Data were acquired using pClamp 10 software, sampled at 50 kHz and filtered at 1 kHz. Analysis was performed with AxoGraphX (Axograph Scientific).

c-Fos Immunostaining: D1-Lrrk2-KO and littermate control mice expressing tdTomato under the Drd1a promoter were injected with either SKF 81297 (2mg/kg) or saline (10 ml/kg) 90 mins

prior to transcardial perfusion with 4% PFA. Brains were removed and post-fixed in 4% PFA overnight at 4 °C. Coronal sections (50 µm) from each treatment and genotype groups were processed in parallel using a vibratome (Ted Pella). Sections were washed in PBS (3 × 10 min) and blocked with 10% normal goat serum for 2 h at RT. Sections were incubated in primary antibody rabbit mAb cFos (1:500, Cell Signaling Technology, #2250S) for 48 h at 4 °C, washed in PBS (3 x 10 min), then incubated in the secondary antibody anti-rabbit Alexa Fluor 488 (1:500, Invitrogen, #A11008) or Goat anti-Rabbit IgG (H+L) Cross-Adsorbed Secondary Antibody, Cyanine5 (1:500, ThermoFisher, #A10523) for 2 h at RT and washed with PBS (1 x 10 min), and 0.1 M PB (1 x 10 min). Slices from the microinjection experiments were additionally incubated with primary antibody Chicken IgY GFP Polyclonal Antibody (1:100, ThermoFisher, #A10262) for 48 h at 4 °C and with secondary antibody Goat anti-Chicken IgY (H+L) Cross-Adsorbed Secondary Antibody, Alexa Fluor Plus 488 (1:100, ThermoFisher, #A32931). Sections were mounted with Vectashield with DAPI (Vector Laboratories).

Drugs and chemicals: Alcohol (Decon Laboratories, 190 proof, glass container) was dissolved in tap water for drinking experiments or ACSF for electrophysiology. Quinine hemisulfate monohydrate (Sigma-Aldrich) was dissolved in the 20% alcohol solution or in tap water. SKF81297, NBQX, CPP, and PKI were purchased from Tocris. SCH23390, gabazine, and kynurenic acid (sodium salt) were obtained from Abcam.

Lrrk2 mRNA expression: FASTQ files were downloaded (GSE81672: Nac, PFC, BLA, vHipp; GSE89692: PFC, VTA and PRJEB36194: DMS) using SRA toolkit. Only naïve C57BL6/J wild type mice were used in this analysis. Read quality, length, and composition were assessed using FastQC before trimming low quality bases (Phred < 20) and adapters using Fastp. Alignment to the GRCm39 Gencode genome assembly and gene-level counts were generated using STAR with default options. BiomaRt in the R (version 4.0.3) was used to calculate FPKM of gene counts.

Stereotaxic virus injections*:* Mice were anesthetized with isoflurane and placed in the stereotaxic apparatus (Kopf Instruments). Adeno-associated viral (AAV) vectors for ChR2 expression (rAAV5-CaMKIIhChR2(H134R)-EYFP; $1.5x10^{13}$, Penn Vector Core), Cre expression (pAAV.CMV.HI.eGFP-Cre.WPRE.SV40) and eGFP expression

(pENN.AAV.CB7.CI.eGFP.WPRE.rBG) were bilaterally injected into DMS (276 nl, from bregma: AP, +1.1; ML, ±1.2; DV, -3.0 mm for ChR2 expression and AP, +0.6, +1.1; ML, ±1.4, ±1.2; DV, -3.2 mm for Cre and eGFP expression) using a Nanoject II (Drummond Scientific).
